# Exposure to Low Doses of Oxybenzone During Perinatal Development Alters Mammary Gland Stroma in Female Mice

**DOI:** 10.3389/ftox.2022.910230

**Published:** 2022-05-20

**Authors:** Klara Matouskova, Jennifer Bugos, Sallie S. Schneider, Laura N. Vandenberg

**Affiliations:** ^1^ Department of Environmental Health Sciences, School of Public Health and Health Sciences, University of Massachusetts, Amherst, MA, United States; ^2^ Baystate Medical Center, Springfield, MA, United States

**Keywords:** 2-hydroxy-4methoxybenzophenone, endocrine disrupting chemical, xenoestrogen, terminal end bud, collagen, white fat

## Abstract

Mammary stroma is a prominent modulator of epithelial development, and a complex set of interactions between these tissue compartments is essential for normal development, which can be either permissive or restrictive in tumor initiation and progression. During perinatal development, exposures of mice to oxybenzone, a common UV filter, environmental pollutant and endocrine disruptor, induce alterations in mammary epithelium. Our prior research indicates that oxybenzone alters mammary epithelial structures at puberty and in adulthood. We had also previously observed changes in the expression of hormone receptors at puberty (e.g., oxybenzone induced a decrease in the number of epithelial cells positive for progesterone receptor) and in adulthood (e.g., oxybenzone induced a decrease in the number of estrogen receptor-positive epithelial cells), and increased body weight in adulthood. Here, we investigated mammary stromal changes in BALB/c animals exposed during gestation and perinatal development to 0, 30, or 3000 μg oxybenzone/kg/day. In mice exposed to 30 μg/kg/day, we observed morphological changes in adulthood (e.g., a thicker periductal stroma and adipocytes that were considerably larger). We also observed an increased number of mast cells in the mammary stroma at puberty which may represent a transient influence of oxybenzone exposure. These results provide additional evidence that even low doses of oxybenzone can disrupt hormone sensitive outcomes in the mammary gland when exposures occur during critical windows of development, and some of these effects manifest in later life.

## Introduction

The etymology of the word stroma comes from the Greek for a “bed” or “layer”. In the mammary gland, the stroma is classified as a “connective tissue” type but neither the role of a “bed” or “connector” conveys the importance of the stroma as it is understood today. Beginning during embryonic development, the mammary gland is established from an interplay between the dermal epithelium and underlying mesenchyme (stroma) (Parmar and Cunha, 2004). In fact, transplantation of mammary epithelium into salivary stroma (and vice versa) illustrates the importance of the stroma for establishing the morphology of the epithelium; when such transplantations occur using fetal tissues, the epithelium takes on the morphological characteristics associated with the stroma where it is transplanted (Sakakura et al., 1976). The same is true in carcinogenesis, where epithelial cells exposed to a carcinogen develop mammary neoplasia only if the stroma was also primed with carcinogen and conversely, normal stroma can prevent a carcinogen-exposed epithelium from tumorigenesis (Maffini et al., 2004).

During normal development, stromal extracellular matrix proteins contribute to epithelial side-branching *via* induction of Wnt pathways and suppression of excessive branching *via* secretion of matrix metalloproteinases (MMPs) and expression of TGFβ (Wiseman and Werb, 2002). Beyond para- and autocrine signaling, the stroma may modulate endocrine responses in estrogen-nonresponsive epithelium (Haslam and Counterman, 1991). Other regular mediators of the hormonal crosstalk among the two tissue compartments in the mammary gland include parathyroid hormone-related peptide (PTHrP) and IGF-1, the primary mediator of the effects of growth hormone (Hovey et al., 2002). Lastly, adipose tissue, vasculature, and immune cells of the stroma can act as either permissive or restrictive towards the spreading of tumor cells, and therefore these stromal cells modulate the metastatic potential of epithelial tumors *via* a complex network of interactions (Khamis et al., 2012; Hussain et al., 2020).

Endocrine disrupting chemicals (EDCs) have been investigated for their impact on the mammary stromal structures and the cellular components of this tissue compartment (Paulose et al., 2015; Vandenberg, 2021). Other research on EDCs has led to the classification of a new group of chemicals, the metabolism disrupting chemicals (MDCs) (Heindel et al., 2017). MDCs can affect energy balance and fat deposition, with effects suggesting that many of these chemicals act directly on adipocyte differentiation, indirectly on central and peripheral organs with a downstream influence on metabolism, on storage of triglycerides, and/or on signaling pathways altering hunger and satiety (Heindel and Blumberg, 2019). MDCs can also induce dysfunction in white adipose and brown adipose tissue (Francis et al., 2021). Some of these chemicals are also deposited in lipids, and their presence in adipose cells may contribute to continued dysfunction in the stromal compartment.

As an example, it has been proposed that the primary target in the mammary gland for the EDC bisphenol A (BPA) is the stroma. During prenatal development, estrogen receptors (ER) are not expressed in the epithelium, thus, during this period of exposure, BPA is most likely acting *via* receptors expressed in the fetal mesenchyme (Richter et al., 2007; Soto et al., 2008; Soto et al., 2013). Furthermore, the transcriptomal profile of BPA compared to ethinyl estradiol (EE2, an estrogenic positive control) also points to its effects predominantly located in the stromal compartment (Wadia et al., 2013).

There is also evidence that estrogenic EDCs can act on the immune cell populations that reside in the mammary stroma. Many of these immune cells express ER and are therefore sensitive to EDCs that are ER agonists or antagonists (Yoshitake et al., 2008; Hessel et al., 2016). In addition to actions *via* the ER, the aryl hydrocarbon receptor (AhR) and the family of peroxisome proliferator-activated receptors (PPAR) modulate immune cell responses resulting in inflammatory and adverse organ conditions (Sabuz Vidal et al., 2021).

Oxybenzone is an EDC commonly used in sunscreens and personal care products, and found in many fabrics, paints, and other consumer goods (Matouskova and Vandenberg, 2022a). Oxybenzone is added to these products to prevent them, or their users, from UV-induced damage. Several recent studies suggest that the mammary gland is a target of oxybenzone exposures. The first study demonstrated that mice exposed during pregnancy and lactation had alterations to their mammary gland epithelial morphology that persisted long after exposures ceased (LaPlante et al., 2018). A second study found that mice exposed to oxybenzone during perinatal development had increased numbers of epithelial structures in adulthood, including the precocious appearance of alveolar buds, differentiated structures which are typically associated with pregnancy (Matouskova et al., 2020). Finally, a third study using a genetically susceptible mouse model found that oxybenzone promoted epithelial tumors in the mammary gland when mice were fed a high-fat diet throughout adulthood (Kariagina et al., 2020).

Here, we examined the effects of perinatal exposure to oxybenzone on the stromal structures and cells in the mouse mammary gland. Results from our study suggest that there are structural impacts of early exposures to oxybenzone on the mammary stroma that persist into adulthood while effects on some immune cell populations might be transient.

## Methods

### Animals

BALB/c mice [UMass colony described and analyzed in (LaPlante et al., 2018)] were housed in polysulfone cages, with at maximum of five mice of the same sex per cage, and provided food (soy-free chow, ProLab IsoDiet, Brentwood, MO) and tap water (in polysulfone bottles with rubber stoppers and metal tops) *ad libitum*. Animals were maintained at the University of Massachusetts, Amherst Morrill Animal Facility, in temperature- and light-controlled conditions (12-h cycles of light and dark). All experimental procedures were approved by the University of Massachusetts Institutional Animal Care and Use Committee.

### Chemical Administration

**FIGURE 1:**
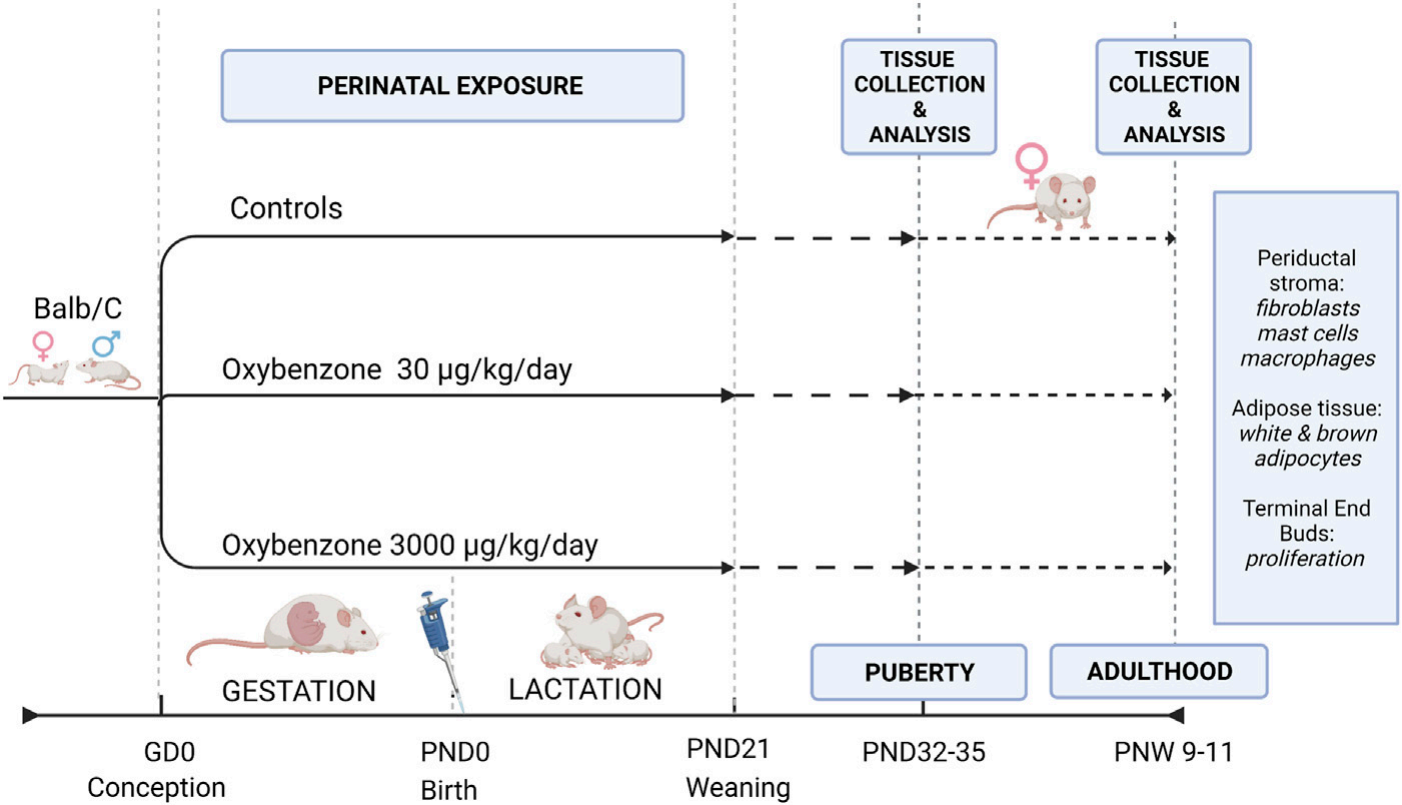
Study Design illustrating the exposure and evaluation periods. GD, gestational day; PND, postnatal day; PNW, postnatal week.

From the first day of pregnancy (when a sperm plug was observed) until the day before weaning (on lactational day 21), the mothers were weighed and orally dosed daily by pipet with either tocopherol stripped corn oil or oxybenzone (Sigma Aldrich, CAS# 131-57-7, certified reference material, purity >99%) dissolved in stripped corn oil. As a result, the offspring evaluated in this study were exposed *in utero* (starting from gestational day 0) and during the first 21 days of postnatal life *via* the mother’s milk. Two doses of oxybenzone were used: 30 μg/kg/day and 3000 μg/kg/day (Figure 1). Depending on the region, season, and individual use of oxybenzone-based personal care product, both doses are likely relevant to human exposures based on back-calculations of urinary concentrations NHANES studies (Woodruff et al., 2011; Han et al., 2016). Furthermore, considering differences in the toxicokinetics between species, both exposures are likely below human exposure levels experienced during sunscreen application (Kariagina et al., 2020; Matouskova and Vandenberg, 2022b). Although the dermal route of exposure results in systemic exposure in humans and rodents, the more controlled approach using an oral route of exposure leads to systemic concentrations of oxybenzone similar to dermal application in mice (Matta et al., 2020; Mutlu et al., 2020).

The animals utilized in this study were assigned to one of the treatment groups based on the treatment their mother received throughout her pregnancy and lactation. Randomization of treatment groups in the maternal animals is described in (LaPlante et al., 2018).

Dams were allowed to deliver naturally, and litters were not culled. Sample sizes for the treatment groups were: controls, 11 litters; 30 μg/kg oxybenzone, 10 litters; 3000 μg/kg oxybenzone, 9 litters. Animals were ear-tagged upon weaning for identification, distributed into separate cages and kept until the peak of puberty (PND 32-35) or adulthood (PND 65-75). Only one female from each litter was selected for each endpoint to avoid litter bias.

### Euthanasia and Tissue Collections

At weaning on PND21, we determined the sex of each offspring and one female from each litter was euthanized at the peak of puberty (PND32-35), or in adulthood (postnatal week 9–11). Results from the dams (the F0 generation) were described (LaPlante et al., 2018). Morphological characteristics of the animals, whole mounts mammary gland and other mammary epithelial endpoints were described previously (Matouskova et al., 2020).

On the day of necropsy, animals were euthanized with CO_2_ inhalation followed by cervical dislocation to ensure death. From every animal we dissected the right fourth inguinal mammary gland, spread it on a glass slide (Thermo Fisher Scientific, Waltham, MA) and fixed it overnight in 10% neutral buffered formalin. The left mammary gland was fixed in 10% neutral buffered formalin overnight before being washed, dehydrated, and embedded in paraffin for histology and immunohistochemistry. The 3rd pectoral mammary glands were collected (avoiding the lymph node), flash frozen on dry ice, and stored at −80°C for qRT-PCR analyses.

### Tissue Embedding, Sectioning, and Histological Staining

Fixed mammary glands were washed in ×1 phosphate buffered saline, dehydrated through a series of alcohols and embedded with paraffin (Leica Biosystems, Richmond, IL) under vacuum. Paraffin blocks were cut into five micrometer sections on a rotary microtome (Fisher Scientific) and mounted on positively charged slides (Fisher Scientific) for histological evaluation and immunohistochemistry.

For histological evaluations, slides were deparaffinized with xylene and a series of alcohols, stained with Harris’ hematoxylin and eosin (Fisher Scientific), dehydrated, and mounted with permanent mounting media (Fisher Scientific). Digital images were collected using a Zeiss Axio Observer. Z1 inverted microscope, a ×10 objective, and a high-resolution color camera (Carl Zeiss Microscopy).

For quantification of periductal collagen fibers, sections were stained with Gomori’s Trichrome Stain (Richard-Allen Scientific/Thermo Fisher Scientific, REF 87020). In short, deparaffinized samples were fixed with Bouin’s fluid (REF# 88038) and stained with Weigert’s Iron Hematoxylin (REF#s 88028 and 88029) and Gomori’s Trichrome Stain (REF# 88030). Individual dyes in the kit stain the collagen fibers blue, cell nuclei are stained black, while cytoplasm and muscle fibers appear red.

### Mast Cells Staining and Analysis

One section from each animal was stained using toluidine blue stain (1% RICCA, Arlington, TX United States). The cover-slipped slides were analyzed using a Zeiss Axio Observer. Z1 inverted microscope, a ×10 objective, and a high-resolution color camera. Each age group required a slightly modified approach as the mammary gland differs at puberty and in adulthood. For adult animals, ten to twelve images of ducts comprised of 20–100 epithelial cells were chosen. For each image, we counted the number of ducts and number of mast cells associated with ducts. For each animal, the ratio of total number of mast cells divided by the total number of ducts was calculated.

The epithelial compartment of the mammary gland is less developed in pubertal mice. Therefore, the ducts selected for analysis were practically all the ducts present in the section. Terminal end buds were excluded from analysis due to their specific character. Also, extra care was taken to *not* include blood vessels in the analysis as they often appear near mammary ducts and their walls contain higher numbers of mast cells.

### Adipocyte Analysis

Two representative images were collected from a mammary gland section stained with H&E from each animal. The selected region was just anterior to the lymph node, focusing on portions of the gland where adipose tissue was the predominant tissue type. Images were collected using a Zeiss Axio Observer. Z1 inverted microscope, ×40 objective, and a high-resolution camera. A nine-point grid was then placed on each image, and the type of fat cell (white vs. brown) and area of each adipocyte at each crosshairs of the grid was measured using the ZEN tool. The number of neighboring fat cells to each adipocyte on the image was counted manually. To avoid bias, a single examinator took all the images, and a different single examinator measured and counted the adipocytes.

### Immunohistochemistry

Expression of two markers, Ki67 and F4/80, was evaluated using standard methods for immunohistochemistry and commercial antibodies including rabbit anti-Ki67 (Fisher Scientific, Cat# RM-9106-S1), a marker of proliferation, and F4/80 (a macrophage marker). Briefly, sections were deparaffinized, hydrated through a series of alcohols, microwaved in 10 mM citrate buffer (pH 6) for antigen retrieval, and treated with hydrogen peroxide to quench endogenous peroxidases. Non-specific binding was blocked with 1% milk protein in 5% normal goal serum (Cell Signaling Technology, Danvers, MA).

Sections were incubated with primary antibodies (typically diluted 1:500 or 1:1000 in blocking solution) at 4°C for 14–16 h. They were then washed and incubated with secondary antibody (goat anti-rabbit, Abcam, Cat# ab64256, undiluted) followed by streptavidin peroxidase complex (Abcam, Cat# ab64269). Diaminobenzidine chromogen (Abcam, Cat# ab64238) was used to visualize reactions. Sections were counterstained with hematoxylin (Fisher Scientific). Each immunohistochemical run included a negative control in which the primary antibody was replaced with 5% normal goat serum.

### qRT-PCR

Total RNA was extracted from the mammary glands using Trizol reagent and a BeadBug microtube homogenizer. We then examined the RNA purity as the A260/A280 ratio and quantified the concentration of RNA in the sample using Nanodrop UV spectrophotometry. 1 mg of RNA from each sample was transcribed to cDNA using reverse transcription. Lastly, 2 µl of cDNA was combined with forward and reverse primers for each target gene (Table 1) and SYBR green master mix to amplify and detect the gene of interest (thermal prolife cycles were set as follows: 10 min at 95°C, 40 cycles of 15 s at 95°C, 30 s at 60°C, and 15 s at 72°C). A melting-curve analysis was conducted to identify nonspecific products. Finally, expression of each target gene was quantified using the ΔΔCt method, which allows each target to be evaluated relative to reference genes. Every sample was run in triplicate for each target gene. Two reference genes were used for the mammary gland analyses: β actin and β2 microglobulin (Table 1). These were averaged for the ΔΔCt quantification.

**TABLE 1:** Primers.

Gene	Forward primer sequence	Reverse primer sequence
β Actin (BA)	CAC​ACC​CGC​CAC​CAG​TTC​GC (89-108)	TTG​CAC​ATG​CCG​GAG​CCG​TT (162-143)
β2 Microglobulin (B2M)	CCG​GCC​TGT​ATG​CTA​TCC​AG	TGT​TCG​GCT​TCC​CAT​TCT​CC
PPARδ	GAT​GGA​AGA​CCA​CTC​GCA​TT	GGA​GCA​GAA​ATG​CTG​GAG​AA
Fatty Acid Synthase (Fas)	TAATTGGCTCCACCAAATCC	CCA​TGC​TCC​AGG​GAT​AAC​AG
αl Collage | (Col1a1)	GGA​GAG​AGC​ATG​ACC​GAT​GG	AAG​TTC​CGG​TGT​GAC​TCG​TG
Fibronectin (F1_7)	ATG​AGA​AGC​CTG​GAT​CCC​CT	GAG​AGC​TTC​CTG​TCC​TGT​CT
Adiponectin (Plin2)	GTG​AAG​ACC​GTG​ACC​TCT​GC	CCC​TTG​CAG​GCA​TAG​GTA​TT

### Statistical Analysis

GraphPad (Prism version 9.1.1) was used to analyze continuous variables. Normally distributed continuous data were evaluated with one-way ANOVA followed by Tukey *post-hoc* tests. For all evaluations, a *p*-value of less than 0.05 was considered statistically significant. All continuous data are graphed as a mean ± standard error. The minimum sample size used across treatment groups was: controls, *n* = 10; 30 μg/kg/day oxybenzone, *n* = 7; 3000 μg/kg/day oxybenzone, *n* = 6. At each age, only a single animal was selected from each litter to avoid litter bias. All analyses were conducted by experimenters blinded to treatment group.

## Results

### The Periductal Stroma is Thicker in Animals Exposed Perinatally to Oxybenzone When Evaluated in Adulthood

**FIGURE 2:**
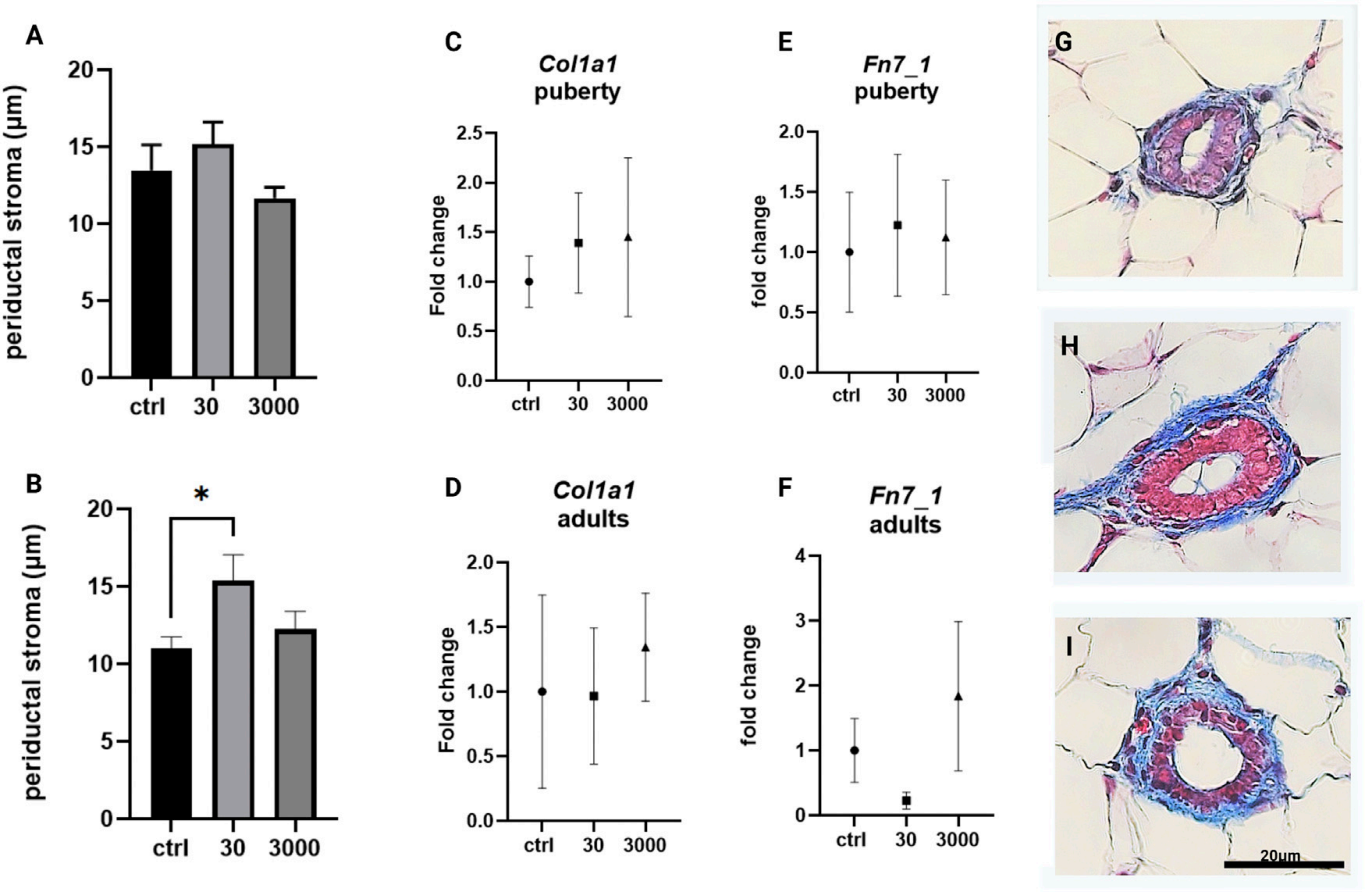
Effects of oxybenzone on periductal stroma, collagen and fibronectin. **(A)** The width of periductal stroma at puberty. **(B)** The width of periductal stroma in adulthood. **(C)** Relative mRNA expression of Col1a1 mRNA at puberty. **(D)** Relative mRNA expression of Col1a1 mRNA in adulthood. **(E)** Relative mRNA expression of Fn7_1 mRNA at puberty. **(F)** Relative mRNA expression of Fn7_1 mRNA in adulthood. **(G–I)** Representative images of periductal stroma in adult animals, in controls **(G)**, and treatment groups of 30 **(H)** and 3000 μg/kg/day **(I)**. Scale bar = 20 µm in each image. * indicates significance <0.05: ANOVA, Tukey posthoc test. All qRT-PCR expression data were normalized using the ΔΔCt method.

We began by measuring the width of the loose stroma, e.g., the periductal stroma, that surrounds the mammary ducts. Of the two doses of oxybenzone tested, 30 μg/kg/day induced the expansion of the periductal stroma in both age groups, although this was only statistically significant in adult animals (Figures 2A,B; ANOVA, *p* values = 0.32 and 0.035 respectively; Tukey posthoc test, control versus 30 μg/kg in adult animals = 0.028).

The loose stroma has a cellular component, the fibroblasts, and a matrix comprised of collagens, glycoproteins, and proteoglycans. We assessed the expression of the gene for type I collagen using primers specific to its pro-α1(I) chain (*Col1a1*), the most abundant collagen type. There was no effect of oxybenzone on expression of *Col1a1* in either pubertal or adult mammary glands (Figures 2C,D; ANOVA, *p* value = 0.835 and 0.879 in pubertal and adults resp.). We also evaluated the expression of fibronectin (*Fn7_1*), a matrix glycoprotein. Although expression of *Fn7_1* was decreased in adult glands collected from mice exposed to 30 µg oxybenzone/kg/day, this effect was not statistically significant (Figures 2E,F, ANOVA *p* values = 0.954 and 0.273).

### Adipocytes in the Mammary Stroma

**FIGURE 3:**
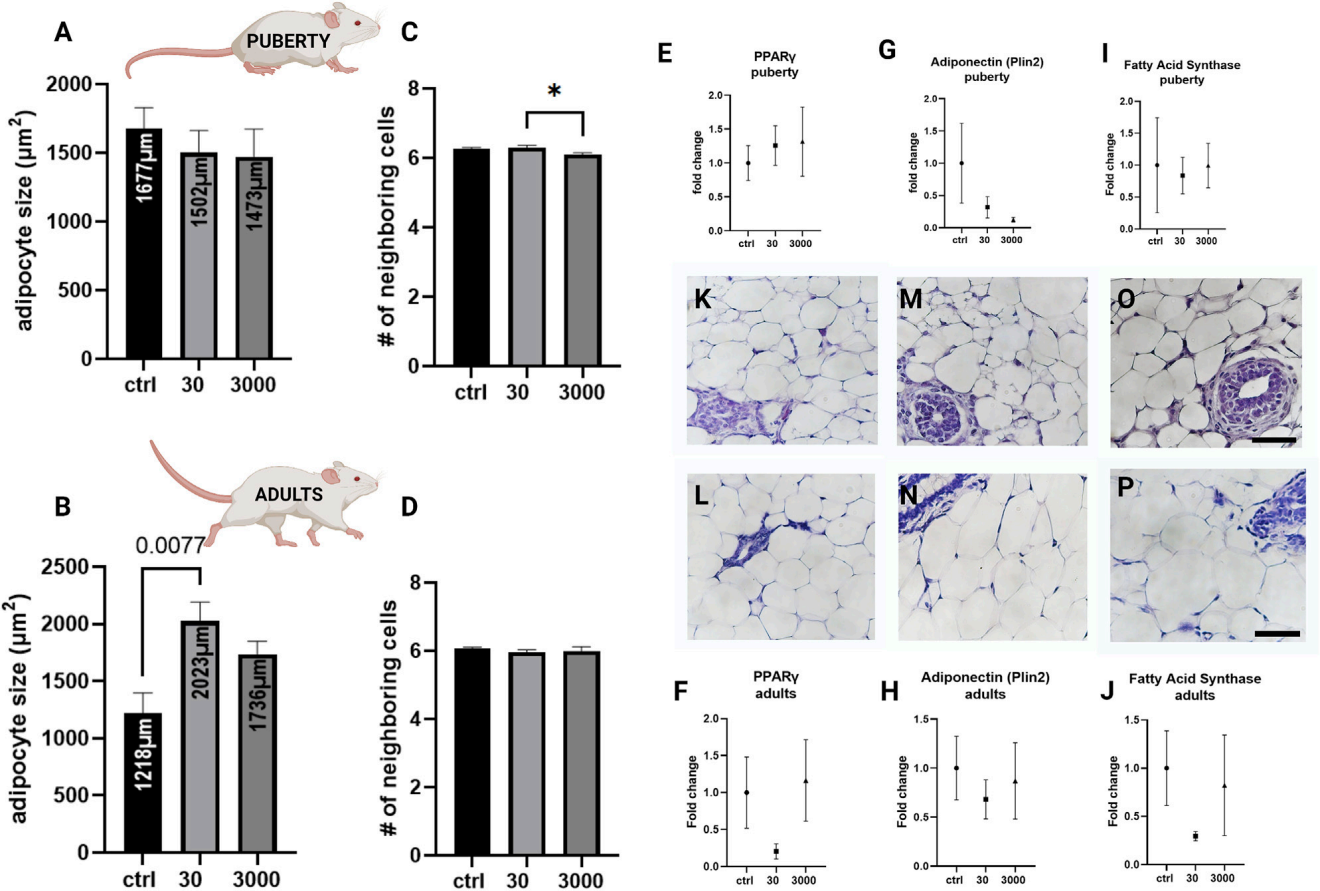
Alterations to white adipose tissue in oxybenzone-treated females. **(A)** Average size of white adipocytes at puberty. **(B)** Average size of white adipocytes in adulthood. **(C)** Number of adipocyte-adjacent cells at puberty. **(D)** Number of adipocyte-adjacent cells in adulthood. **(E)** Relative mRNA expression of PPARγ mRNA at puberty **(F)** Relative mRNA expression of PPARγ mRNA in adulthood. **(G)** Relative mRNA expression of Plin2 mRNA at puberty. **(H)** Relative mRNA expression of Plin2 mRNA in adulthood **(I)** Relative mRNA expression of Fas mRNA at puberty **(J)** Relative mRNA expression of Fas mRNA in adulthood; **(K,M,O)** Representative images of adipose tissue in pubertal animals in controls **(K)** and treatment groups of 30 **(M)** and 3000 μg/kg/day **(O)**. **(L,N,P)** Representative images of adipose tissue in adult animals in controls (L), 30 (N), and 3000 μg/kg/day **(P)**. Scale bar = 20 µm in each image.* indicates significance <0.05: ANOVA, Tukey posthoc test. All qRT-PCR expression data were normalized using the ΔΔCt method.

Next, we assessed the size, organization, and type of adipocytes in the mammary stroma. In the white adipose tissue compartment, at puberty, the white adipocytes were not significantly different in size between the treatment groups (Figure 3A, *p* value = 0.641, ANOVA). However, we observed that adult adipocytes were on average almost two times larger in response to oxybenzone (30 μg/kg/day) compared to controls (Figure 3B, ANOVA, *p* value = 0.0084, Tukey posthoc = 0.077). Furthermore, between puberty and adulthood, the adipocytes in control mammary glands decreased in size by about 1/3 (from an average of 1677 μm^2^–1218 μm^2^; compare 3A and 3B). In contrast, white adipose cells from oxybenzone-exposed animals *increased* in size in the mammary gland from 1502 to 2023 μm^2^ in the 30 μg/kg/day group, and from 1473 to 1736 μm^2^ in the 3000 μg/kg/day group.

To determine if oxybenzone treatment altered other aspects of adipocyte biology, we also examined the number of fat cell neighbors for adipocytes within the mammary adipose tissue. The count is a measure of the cellular organization and packing of adipocytes in the tissue. There was a modest but statistically significant difference between the two oxybenzone treated groups at puberty, suggesting that the higher dose of oxybenzone might be producing subtle effects on the organization of the cells in the fat pad. Although there were modest shifts in this measure, overwhelmingly the adipocytes are organized in hexagons (Figures 3C,D; ANOVA, *p* value = 0.041 at puberty, Tukey posthoc = 0.0491; ANOVA, *p*-value = 0.6 in adulthood).

We next selected three genes representing different aspects of adipocyte function and evaluated their expression. The transcription factor and abundant fat protein, PPARγ, is critical for adipogenesis. Although we saw PPARγ downregulation in the adult mammary gland following exposure to 30 μg/kg/day oxybenzone, this difference was not statistically significant (Figures 3E,F; ANOVA, *p* values = 0.798 and 0.286). Adiponectin is an adipocyte-released cytokines mediating metabolism; adiponectin stimulates β oxidation of fatty acids. We saw no significant effects of oxybenzone on adiponectin expression in either the pubertal or the adult mammary gland (Figures 3G,H; ANOVA, *p* values = 0.501 and 0.732). Finally, fatty acid synthase (Fas) is involved in the biosynthesis of fatty acids. Again, we saw a non-significant downregulation of Fas in adult animals from the 30 μg/kg/day oxybenzone exposure group (Figure 3J; ANOVA, *p* value = 0.299).

**FIGURE 4:**
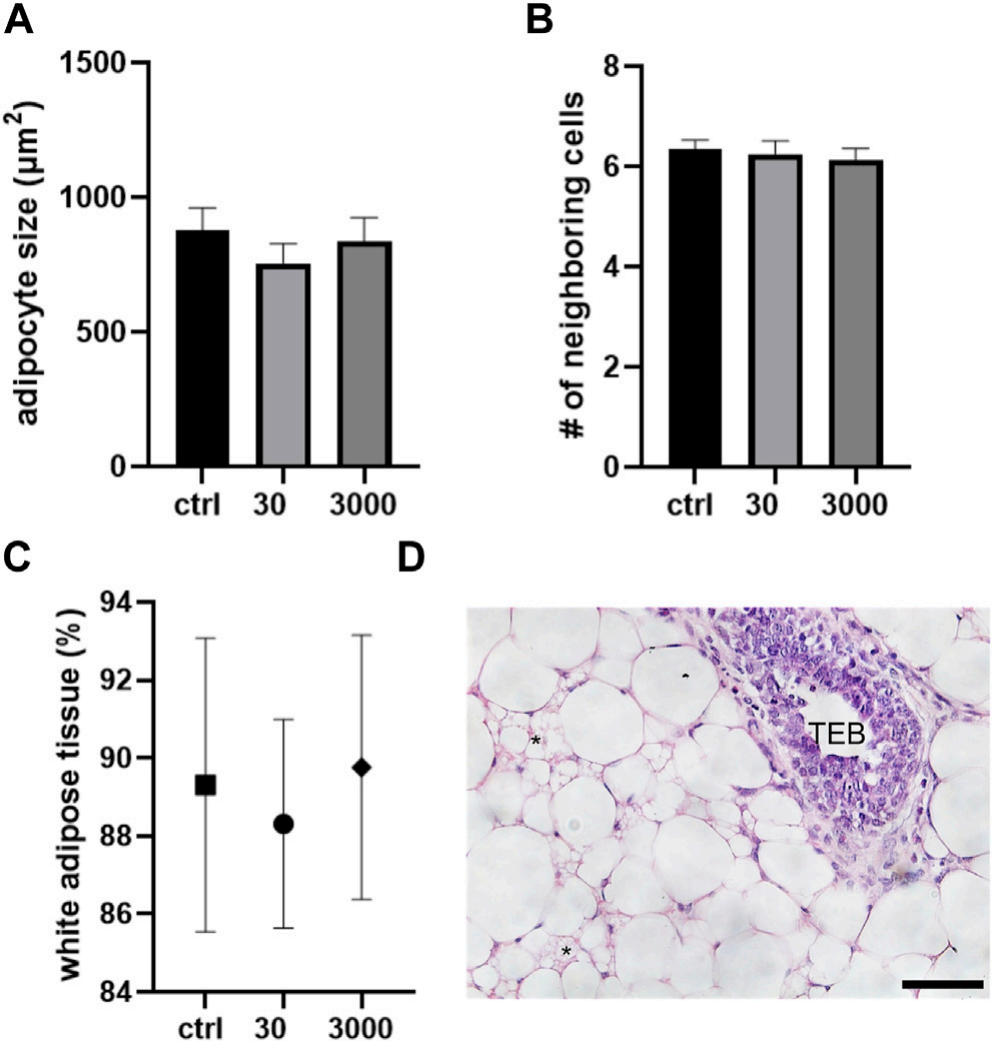
Oxybenzone and brown adipose tissue at puberty. **(A)** Size of brown adipocytes by treatment group. **(B)** Number of neighboring cells in the brown adipose tissue. **(C)** Percent of the mammary stroma comprised of white adipocyte tissue. **(D)** White and brown adipose tissue in the mammary fat pad. * indicates brown adipocytes. The epithelial structure in this image is a TEB. Scale bar = 20 μm.

In addition to its white adipose tissue, the pubertal mammary gland bears occasional areas of brown fat. Brown adipocytes are smaller than white fat cells (averaging 900 vs. 1677 μm^2^) with multiple lipid droplets apparent within each cell. In our analyses of the brown fat cells in the pubertal mammary gland, we saw no effect of perinatal oxybenzone exposures on the size of brown fat cells in mammary glands collected at puberty (Figure 4A; ANOVA, *p* value = 0.54). In all three treatment groups, the brown adipocytes maintained their hexagonal arrangement (Figure 4B).

Lastly, we investigated five non-overlapping regions of the mammary gland and compared the proportions of white vs. brown adipocytes. Perinatal oxybenzone exposure did not alter the proportion of the fat pad that is comprised of white adipose cells (Figure 4C). In all treatment groups, the pubertal mammary gland in BALB/c female mice is comprised of about 90% of white and 10% of brown adipocytes.

### Macrophages and Mast Cells in the Oxybenzone-Treated Mammary Gland

**FIGURE 5:**
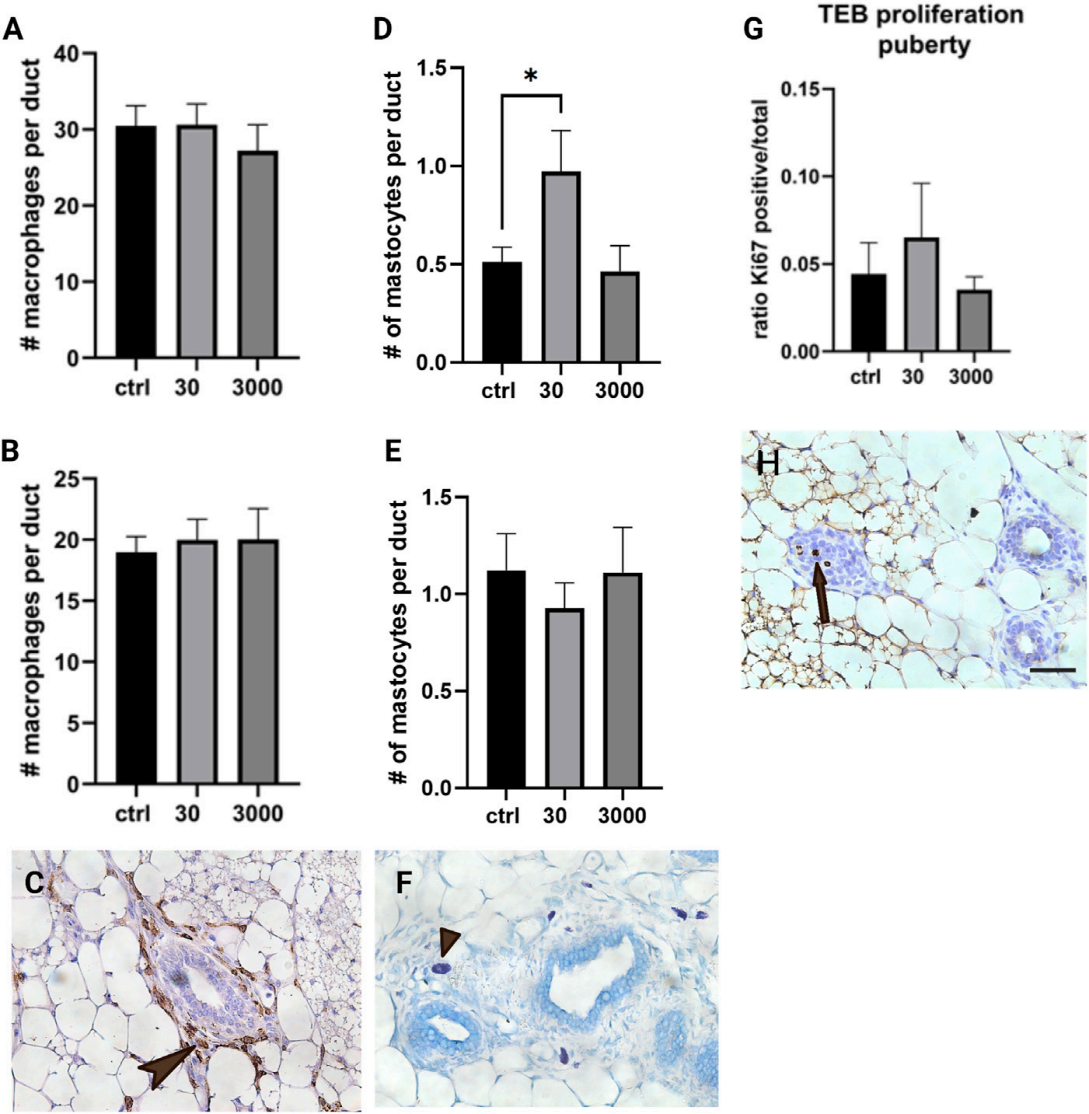
Effect of oxybenzone on macrophages, mast cells, and proliferation in TEBs. **(A)** Number of macrophages in the periductal stroma at puberty. **(B)** Number of macrophages in the periductal stroma in adulthood. **(C)** Immunohistochemistry for F4/80, a marker of macrophages. This image illustrates macrophages localized in the mammary periductal stroma (arrowhead). **(D)** Number of mast cells in periductal stroma at puberty. **(E)** Number of mast cells in periductal stroma in adulthood. **(F)** Toluidine blue staining, highlighting mast cells in the mammary stroma (staining purple, arrow tip). **(G)** Ratio of Ki67 positive cells to total cells in TEBs at puberty. * indicates significance, *p* < 0.05, ANOVA with Tukey posthoc test. **(H)** Proliferation in a TEB as indicated by immunohistochemistry for Ki67 (three cells in mitosis, the arrow pointing towards the middle proliferating cell). Scale bar = 20 μm.

Scattered throughout the stromal matrix are immune cells that are responsible for maintaining the health of the mammary gland. Using slides stained with F4/80 antibody, we quantified the number of macrophages present in the mammary stroma. Macrophages are ample in the periductal stroma in both ages, averaging about 30 per duct at puberty and 19 per duct in adult animals. The number of macrophages in the periductal stroma was not altered by perinatal oxybenzone exposure at either of the ages we evaluated (Figures 5A–C; ANOVA, *p* value 0.672 at puberty, and 0.854 in adulthood).

We also evaluated the number of mast cells localized to the periductal stroma. Mast cells were less abundant than macrophages, with an average of 0.51 per mm^2^ at puberty and 1.12 per mm^2^ in adult animals. We observed an almost doubling of the number of mast cells in females from the 30 μg/kg/day group when the evaluation was conducted at puberty (Figure 5D; ANOVA, *p* value = 0.033, Tukey posthoc = 0.047); however, this effect appears to be transient because no differences were observed in adulthood (Figure 5E; ANOVA, *p* value = 0.73).

### Proliferation in the Terminal End Bud Structures

Earlier studies suggest that mast cells are required for normal proliferation in the epithelium as well as in the terminal end buds (TEBs) specifically (Lilla and Werb, 2010). After seeing the number of mast cells increase in response to 30 μg/kg/day oxybenzone (Figure 5F), we next quantified proliferation specifically in the TEB structures in the pubertal mammary gland. The number of cells in the TEB expressing Ki67, a marker of proliferation, was not affected by perinatal oxybenzone exposure (Figure 5G, ANOVA *p* value = 0.64). These results are consistent with the lack of an effect of oxybenzone on the size of the average TEB in the pubertal mammary gland (data not shown, ANOVA, *p* value = 0.97).

## Discussion and Conclusion

In our prior work, we reported on the mammary epithelial features in BALB/c mice exposed to oxybenzone during gestation and the perinatal period (Matouskova et al., 2020). We observed effects of oxybenzone on the morphology of the epithelial structures at puberty (e.g., more TEBs and a longer ductal extension) and in adulthood (e.g., a higher volume fraction of alveolar buds in oxybenzone-exposed females). We also observed changes in the expression of hormone receptors at puberty (e.g., oxybenzone induced a decrease in the number of PR-positive epithelial cells) and in adulthood (e.g., oxybenzone induced a decrease in the number of ER-positive epithelial cells). The results of the current study suggest that these effects in the epithelium are occurring concurrently to alterations to the stroma, and provide additional areas to probe the mechanisms by which oxybenzone alters mammary gland development.

We report here subtle but significant effects of oxybenzone on stromal tissue organization, e.g., the periductal collagen layer was almost 50% wider in adult animals exposed to the lower dose of oxybenzone (Figure 2B). However, expression of two specific matrix components, collagen type 1 and fibronectin, were not significantly affected. These results may be due to the scope of the oxybenzone-induced alteration which is localized to the periductal stroma and therefore may be challenging to detect in RNA isolated from the entire gland. It is also plausible that a type of collagen fiber other than collagen one or the cross-linking and organization of the collagen, two aspects not evaluated in the current study, are altered by oxybenzone. Several other glycoproteins in the mammary extracellular matrix such as laminin and tenascin, as well as TGFβ and hepatocyte growth factors mediate growth of the matrix (Schedin et al., 2004). Because activation of fibroblasts leads to increased extracellular collagen, fibronectin, and laminin deposition and ultimately to alterations in the periductal stroma (Vafaizadeh et al., 2010), additional studies should continue to characterize the effects of oxybenzone on mammary fibroblasts.

On the cellular and tissue levels, exposure to 30 μg/kg/day of oxybenzone during gestation and perinatal development altered the mammary adipose tissue in adulthood (Figure 3). In controls, the size of adipocytes decreased from nearly 1700 to 1218 μm^2^ between puberty and adulthood. Yet, in response to developmental exposure to oxybenzone, adult adipocytes not only reversed the direction and grew larger, but they also almost doubled in size (from 1218 to 2023 μm^2^ in the 30 μg/kg/day group). The hypothesis that EDCs could play a part in metabolism, adipocyte biology, and obesity was proposed by Grun and Blumberg (2006), and they coined the term obesogens for chemicals that promote obesity in humans and animals. According to the obesogen hypothesis, these chemicals act by altering adipocytes directly and indirectly by increasing their number and storage of triglycerides, changing basal metabolism and energy balance in favor of storage of calories, and modifying food intake through effects on other organs (Heindel et al., 2017).

Oxybenzone is a well-recognized ER-agonist (Watanabe et al., 2015; Balázs et al., 2016) and other estrogenic chemicals have distinct effects on adipocytes isolated from different parts of the body; for example, the effects of bisphenols on the transcriptome of omental fat cells differs significantly from their effects on the transcriptome of subcutaneous adipose cells (Peshdary et al., 2020). A recent interlaboratory study used the 3T3-L1 pre-adipocyte model cell line and found that triglyceride accumulation was induced by several EDCs (Kassotis et al., 2021). Future studies using both cell cultures and animal models would enable us to evaluate the mechanisms that may lead to disruption of the adipose tissue in oxybenzone-treated animals.

Above all other players, PPARγ is described as both a necessary and sufficient factor of adipogenesis in mammals and fish (Wafer et al., 2017) due to its ability to stimulate differentiation of pre-adipocytes to adipocytes. Therefore, PPARγ is considered the mediator of adipocyte hypertrophy (Kubota et al., 1999). While our analyses did not detect significant differences in relative mRNA expression of PPARγ, adiponectin, or fatty acid synthase (Figures 3E–J), prior studies using human bone marrow-derived mesenchymal stem cells demonstrated that both oxybenzone and its metabolite benzophenone eight increases the size and number of lipid droplets in differentiated adipocytes (Shin et al., 2020). In mesenchymal stem cells, Shin et al. also demonstrated that oxybenzone binds PPARγ and significantly increases expression of PPARγ mRNA (but not PPARα, PPARβ, adiponectin or fatty acid binding protein 4). Additional studies using high-throughput approaches are needed to understand the full complement of receptors that are bound by oxybenzone, either as an agonist or an antagonist.

**FIGURE 6:**
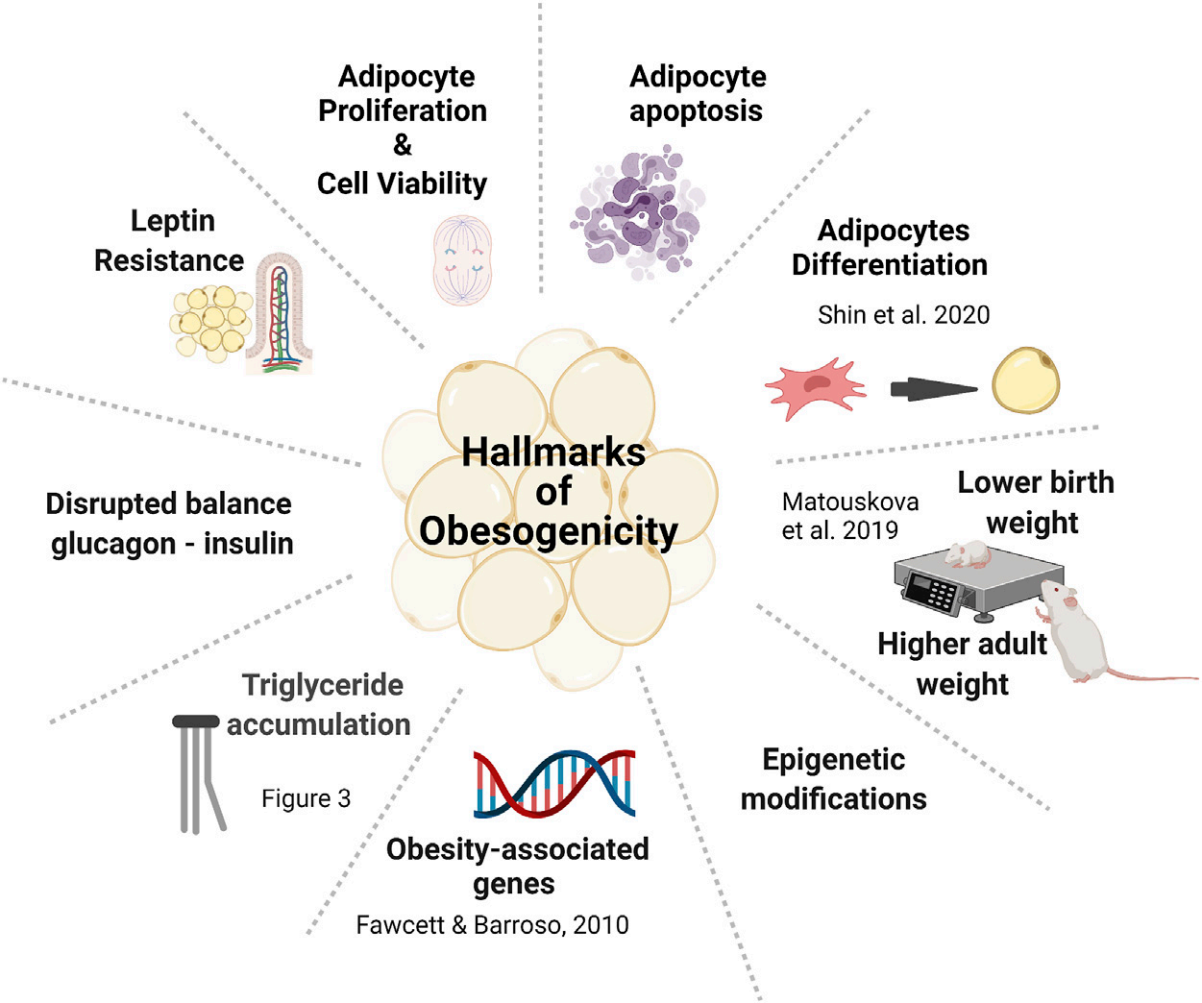
Hallmarks of obesogenesis for oxybenzone.

Based on prior studies and our evaluations in mice, oxybenzone is a suspected MDC. While there is a genetic component to obesity (Fawcett and Barroso, 2010), non- Mendelian aspects of obesogenesis are ever more appreciated. Figure 6 summarizes features of obesogenesis independent of genetic inheritance such as adipocyte differentiation, proliferation and apoptosis, and disruption of metabolism related hormones [e.g., the balance between insulin and glucagon or leptin, as demonstrated for BPA (Angle et al., 2013)] relevant for oxybenzone. In humans, parental obesity begets offspring obesity *via* persistent lifestyle habits (Gray et al., 2018) but emerging hypotheses also point to possible epigenetic transgenerational inheritance of obesity due to maternal diet and possibly also due to exposures to environmental contaminants (Lillycrop, 2011; Chamorro-Garcia et al., 2013). Additional studies are also needed to investigate whether adipose tissues have “windows of sensitivity” to oxybenzone and other MDCs, as well as the span between an exposure and effect. The *in utero* period seems the most likely window of vulnerability, however, these windows may differ by chemical (Heindel and Blumberg, 2019).

Similar effects as seen here (hypertrophy of adipocytes in adulthood) have also been shown following exposures to low doses of other ERα agonists including EDCs such as dioxins and PCBs, and also high-fat diet (Davis et al., 2013; Le Magueresse-Battistoni, 2020). It has been suggested that unlike hyperplasia (an increase in the number of adipocytes), hypertrophy is a sign of dysfunctional development of the adipocyte cells. Moreover, expanded adipocytes can lead to cellular hypoxia and chronic sub-inflammation along with an increased influx of immune cells and local increase of reactive oxygen species (Bokobza et al., 2021). Therefore, markers of inflammation, and possibly, the mechanisms by which inflammation in the mammary stroma affect the long-term health of the epithelium should be evaluated in future research studies.

We observed an increase in the number of mast cells at puberty associated with oxybenzone exposure, but this increase was no longer observed in adulthood, suggesting that this effect may be transient (Figure 5). The changeable number of mastocytes in the mammary gland is not unexpected as these cells express ER and respond to ER agonists (Jing et al., 2012). Mast cells promote ductal elongation during development, corresponding to their role as promoters of angiogenesis and tumor cell proliferation (Ranieri et al., 2009). Even transient effects on the mast cell population may therefore affect the structure of the mammary gland and its resilience to cancerous progression although the exact role of mast cells in breast cancer remains unresolved (Aponte-Lopez et al., 2018). The pubertal increase in the number of mast cells in the periductal stroma might be a consequence of the increased area of the periductal stroma, however, we observed no effect on the population of macrophages in the same tissue compartment. Alternatively, the increase might be due to a chemokine-dependent difference established during exposure and manifested in the pubertal hormonal milieu. Our future studies will expand these evaluations to other immune cell populations that are important to the health of the mammary gland at different stages of development.

The doses used in this study were selected to be relevant to human real-life exposures. Other studies such as those conducted by the National Toxicology Program have administered much higher doses (e.g., concentrations of 1000–50,000 ppm of oxybenzone in feed) (Nakamura et al., 2015; Mutlu et al., 2017). While we have seen most effects at the lower dose (30 μg/kg of body weight, administered daily to the mothers of the offspring in our experiment throughout pregnancy and lactation), it is difficult to determine based on the two dose groups that we have evaluated whether the effects we have observed are consistent with non-monotonic dose responses, e.g., effects that are greater at lower doses than at higher doses. However, it has been previously demonstrated for other EDCs that effects observed after exposures to low doses cannot be extrapolated from high exposures and vice versa (Zoeller et al., 2012; Demeneix et al., 2020). Furthermore, in our own prior study of oxybenzone, we observed a decrease in anogenital distance in male mice exposed to 30 or 212 μg/kg/day, but no effect on anogenital distance in males exposed to 3000 μg/kg/day (Matouskova et al., 2020). Although we are not aware of any studies of oxybenzone that have examined a larger number of doses, the effects we have observed in the lower dose group should not be dismissed because of the absence of effects after exposures ×100 higher (Vandenberg, 2019). Endogenous hormones produce profound effects in nano- and picomolar concentrations that are also not observed at higher concentrations, suggesting a plausible biological explanation for non-linear responses in endocrine-mediated outcomes (Vandenberg et al., 2012).

The effects of environmental chemicals on the mammary gland are often poorly evaluated, if they are evaluated at all, in studies used by regulatory agencies for safety determinations (Cardona and Rudel, 2020). Unfortunately, the composition and health of the mammary gland stroma is not evaluated as an adverse effect and in the absence of those data, chemicals causing subtle but consequential effects on this tissue compartment might be falsely concluded as having no negative effect on human health. Malfunctioning periductal stroma may permit cancer initiation or invasive activity of breast cancer cells. Higher adipogenicity enhances storage of fat-soluble environmental chemicals in the mammary gland and their role in development of the mammary gland cannot be overlooked (Brenot et al., 2020). Improving evaluations of mammary gland health in regulatory studies will likely have an important effect on public health protections.

In conclusion, we have demonstrated here that the stroma of the mammary gland is a sensitive target of oxybenzone, a UV filter commonly used as a sunscreen in personal care products, packaging of perishable foods and drinks, and in paints, textiles, and plastics. Our data suggest that perinatal exposure to low doses of oxybenzone induces adipocyte hypertrophy and expands the periductal stroma in adulthood. Perinatal exposures to oxybenzone also affect mast cell populations in the mammary gland at puberty, but not in adulthood, suggesting these effects may be transient. Additional work is needed to fully elucidate the mechanisms responsible for these alterations and to better understand their possible correlations with adverse clinical conditions.

## Data Availability

The original contributions presented in the study are included in the article/Supplementary Materials, further inquiries can be directed to the corresponding author.
